# Financial Conflicts of Interest Among the Authors of the Clinical Practice Guidelines for Rheumatoid Arthritis in Japan

**DOI:** 10.7759/cureus.46650

**Published:** 2023-10-07

**Authors:** Anju Murayama

**Affiliations:** 1 School of Medicine, Tohoku University, Sendai, JPN; 2 Population Health Science and Policy, Icahn School of Medicine at Mount Sinai, New York, USA

**Keywords:** rheumatoid arthritis, public health policy, evidence-based medicine (ebm), evidence-based guidelines, medical ethics, clinical practice guideline, conflicts of interest

## Abstract

Objective

To assess the financial relationships between pharmaceutical companies and authors of the 2020 Japan College of Rheumatology Clinical Practice Guidelines (CPG) for the Management of Rheumatoid Arthritis and to evaluate the quality of evidence supporting the guideline recommendations.

Methods

This retrospective study evaluated financial relationships between all 27 authors of the CPG and pharmaceutical companies in Japan. Personal payments from pharmaceutical companies to these authors between 2016 and 2020 were extracted from publicly disclosed databases for each pharmaceutical company. The quality of the evidence supporting the CPG recommendations was also assessed.

Results

All 27 authors received personal payments from pharmaceutical companies, totaling $3,683,048 over five years. The median and mean payments per author were $101,624 and $136,409, respectively. Speaking compensations accounted for more than 80% of all personal payments. More than 77.8% (21 authors), 66.7% (18 authors), and 51.9% (14 authors) received more than $10,000, $50,000, and $100,000 in total payments over the five years, respectively. Nevertheless, these financial relationships between the CPG authors and pharmaceutical companies were not disclosed. More than 81.8% of the CPG recommendations were supported by low- or very-low-quality evidence. Of the strong recommendations, 66.7% were supported by low- or very-low-quality evidence.

Conclusion

Even though all CPG authors received substantial amounts of personal payments from pharmaceutical companies, these conflicts of interest (COIs) were not disclosed in the CPG. These findings underscore the urgent need for policy interventions to enhance transparency, integrity, and reliability in the development of CPGs in Japan.

## Introduction

Clinical practice guidelines (CPGs) are instrumental in standardizing evidence-based diagnostic and treatment protocols in rheumatology [1]. However, the integrity of these guidelines can be compromised by conflicts of interest (COIs), both financial and non-financial [2-4]. There are widespread financial relationships between healthcare professionals and the pharmaceutical industry worldwide [5-11]. Over the last two decades, several violations of patient-centered care by CPGs, as well as harmful effects on patients [4,12], have resulted in the establishment of more stringent COI management policies through global collaborations [1,13,14]. Given the increasing influence of CPGs on patients, healthcare professionals, and other stakeholders, rigorous COI management, including full disclosure of COIs, efforts to minimize COIs among CPG authors and organizations, and balanced recommendations by multiple experts, is essential for establishing trustworthy CPGs and promoting patient-centered care [13-19].

Over the past 30 years since the first introduction of methotrexate for rheumatoid arthritis treatment, there has been remarkable development in its systematic treatment strategies [20]. In addition, the success of the first biologic therapy, namely tumor necrosis factor (TNF) inhibitors, has provided further impetus for the development of other biologics targeting different pathways, such as IL-1 inhibitors, IL-6 inhibitors, IL-17 inhibitors, cytotoxic T lymphocyte-associated antigen-4 (CTLA-4) blockers, and Janus kinase (JAK) inhibitors [21]. Due to fierce competition among pharmaceutical companies, the global market for rheumatoid arthritis was expected to be worth approximately US $26 billion in 2019, and pharmaceutical companies have increasingly marketed their products to physicians involved in treating and managing patients with rheumatoid arthritis [8,22,23]. Given these contexts, the authors of the rheumatoid arthritis CPG should properly manage their COI with pharmaceutical companies. This retrospective analysis aimed to assess the financial relationships between pharmaceutical companies and CPG authors using publicly disclosed payment data in Japan.

This article was previously posted to the Authorea preprint server on September 21, 2023.

## Materials and methods

Study setting and participants

This retrospective analysis of personal payments disclosed by pharmaceutical companies evaluated the size and prevalence of financial relationships between pharmaceutical companies and the authors of the CPG for rheumatoid arthritis in Japan. All authors of the 2020 Japan College of Rheumatology Clinical Practice Guidelines (2020 JCR CPG) for the Management of Rheumatoid Arthritis were considered for this study. The Japan College of Rheumatology (JCR), established in 1957, is the sole and most prestigious medical professional society in the field of rheumatology in Japan, boasting 9,860 members as of March 2021. The CPGs published by the JCR wield significant influence in Japan.

Data collection and payment source

The 2020 JCR CPG for rheumatoid arthritis and associated official institutional webpages provided data on author names, gender, affiliations, and positions. Additionally, individual author COI statements and recommendations were extracted from the CPG. As previously described [24-28], all pharmaceutical companies affiliated with the Japan Pharmaceutical Manufacturers Association disclose their payment data concerning speaking, writing, and consulting on their company web pages. However, these companies update this payment data annually and remove data from previous years. Therefore, payment data for the CPG authors between 2016 and 2019 was extracted from a publicly accessible payment database maintained by the Medical Governance Research Institute [29]. Payments to the CPG authors in 2020 were collected directly from each pharmaceutical company's webpage. When necessary, the author contacted the JCR’s development committee and professional offices responsible for publishing these guidelines for further information on the development process and COI statements and policies.

The level of evidence and strength of recommendation was determined by the CPG authors using the Grading of Recommendations, Assessment, Development, and Evaluations (GRADE) methodology [30]. Considering eight factors, i.e., the risk of bias, imprecision, inconsistency, indirectness, publication bias, large magnitude of effect, dose-response gradient, and all residual confounding that would decrease the magnitude of effect, the level of evidence was classified into four categories: strong, moderate, low, and very low, as in many CPGs [31-34].

Analysis

The author conducted a descriptive analysis of the demographic and payment data. Additionally, this study evaluated payment concentrations using the Gini index, as previously described [26,31,35]. Payment values were converted from Japanese yen to U.S. dollars using the 2020 average monthly exchange rate of 106.8 yen per $1. No institutional review board approval was required, as this study only analyzed publicly available data.

## Results

The demographic characteristics of the CPG authors are described in Table 1. Among the 27 CPG authors of the 2020 JCR CPG for rheumatoid arthritis, 19 (70.4%) were male, 22 (81.5%) were affiliated with universities or university teaching hospitals, 9 (33.3%) were university professors, and 18 (66.7%) specialized in rheumatology and immunology. Only one author represented a rheumatoid arthritis patient organization. The median h-index and the number of academic publications were 21 (interquartile range (IQR): 10.5-31.0) and 95 (IQR: 35-161), respectively.

**Table 1:** Demographic characteristics of CPG authors for rheumatoid arthritis in Japan The median and IQR of the h-index and the number of publications represent the median and IQR of these variables among all CPG authors. CPG: Clinical practice guideline, IQR: Interquartile range

Variables	Number of authors, n (%)
Gender	
Male	19 (70.4)
Female	8 (29.6)
Affiliation	
University and university teaching hospitals	22 (81.5）
Professor	9 (33.3）
Other positions including associate professors, assistant professors, and lecturers	13 (48.1)
Other general hospitals	4 (14.8）
Patient organization	1 (3.7）
Specialty	
Rheumatology and clinical immunology	18 (66.7)
Epidemiology, public health, and methodology	3 (11.1）
Orthopedic surgery	3 (11.1)
Pediatrics	1 (3.7)
Rehabilitation medicine	1 (3.7)
Patient	1 (3.7)
Academic performance	
Median h-index (IQR)	21 (11‒31)
Median number of publications (IQR)	95 (35‒161)

Table 2 shows the personal payments from pharmaceutical companies to the CPG authors. All 27 authors received at least one personal payment from pharmaceutical companies between 2016 and 2020. A total of 3,951 payments, amounting to $3,683,048, were made to the CPG authors by 52 pharmaceutical companies over the course of five years. Speaking compensation accounted for 83.9% of the personal payments ($3,089,971) while consulting payments made up 10.3% ($380,217) of the total. The median and mean payments per author were $101,624 (IQR: $16,221-$184,160) and $136,409 (standard deviation (SD): $156,736), respectively. The median and mean annual personal payments per author were $21,085 (IQR: $5,402-$37,968) and $29,000 (SD: $32,035), respectively. The Gini index for per-author personal payments was 0.55. One author received $686,024 in personal payments over five years. More than 77.8% (21 authors), 66.7% (18 authors), and 51.9% (14 authors) received more than $10,000, $50,000, and $100,000 in total payments over the five years, respectively. Although the CPG stated that all authors had self-declared their financial COI status to the JCR and that a COI management committee of the JCR had confirmed all authors with financial COIs as eligible for CPG development, this self-declared COI information was not disclosed in the main text of the CPG nor on the JCR's website. Therefore, we could not evaluate the accuracy of the authors' self-declared COI status.

**Table 2:** Summary of personal payments from pharmaceutical companies to the authors of the CPG for rheumatoid arthritis issued by the JCR ^a ^Percentage of authors with payments representing the proportion of the authors receiving specific amounts of personal payments out of all authors. ^b ^Percentage of payments representing the percentage of a particular type of payment in the total payments. CPG: Clinical practice guideline, JCR: Japan College of Rheumatology

Variables	Value
Total amount of payments, $	3,683,048
Mean per author (standard deviation), $	136,409 (156,736)
Median per author (interquartile range), $	101,624 (16,221‒184,160)
Range, $	187‒686,024
Authors with payments, n (%)^a^	
Any payments	27 (100)
>$10,000	21 (77.8)
>$50,000	18 (66.7)
>$100,000	14 (51.9)
>$250,000	5 (18.5）
>$500,000	1 (3.7）
Type of payments, $ (%)^b^	
Speaking	3,089,971 (83.9)
Consulting	380,217 (10.3)
Writing/Honoraria	180,013 (4.9)
Other	32,848 (0.9)
Gini index	0.55

Regarding the CPG recommendations, a total of 55 unique recommendations were listed. Of these, 15 (27.3%) were strong recommendations, and 40 (72.7%) were conditional recommendations. More than 81.8% (45 out of 55 recommendations) were supported by low- or very-low-quality evidence. Of the 15 strong recommendations, 66.7% (10 out of 15) were supported by low- or very-low-quality evidence, while 87.5% of conditional recommendations were based on low- or very-low-quality evidence (Table 3).

**Table 3:** Evidence level and strength of recommendation underlying the CPG for rheumatoid arthritis in Japan The percentage of recommendations represents the proportion of specific recommendations to the total number of recommendations. CPG: Clinical practice guideline

Level of evidence	Strength of recommendation, n (%)		Total
	Class 1 (strong)	Class 2 (weak and conditional)	
High	3 (5.5)	1 (1.8)	4 (7.3)
Moderate	2 (3.6)	4 (7.3)	6 (10.9)
Low	4 (7.3)	3 (5.5)	7 (12.7)
Very low	6 (10.9)	32 (58.2)	38 (69.1)
Total	15 (27.3)	40 (72.7)	55 (100)

## Discussion

The present study aimed to investigate the financial conflicts of interest among the authors of the 2020 JCR CPG for the management of rheumatoid arthritis. To the best of the author's knowledge, this study is the first to evaluate the prevalence and magnitude of financial relationships between CPG authors and pharmaceutical companies in Japan in the field of rheumatology. Remarkably, all 27 CPG authors received personal payments from pharmaceutical companies between 2016 and 2020, totaling $3,683,048. The median and mean payments per author were substantial, and one author received as much as $686,024 over five years. These findings raise ethical concerns, especially considering that the COI information was not publicly disclosed in the CPG or on the JCR's website. Additionally, more than 80% of recommendations were supported by low- or very-low-quality evidence, including single observational studies, case reports, and the opinions of the CPG authors.

The high prevalence of financial COIs among the authors in this study aligns with previous research, albeit with a higher percentage. This could indicate a field-specific vulnerability to financial COIs, particularly in the realm of rheumatology in Japan. For example, the author previously reported that the prevalence of CPG authors receiving personal payments from pharmaceutical companies was 87.0% in infectious diseases [36], 88% in nephrology [18], 88.2% in gastroenterology [31], 88.6% in urology [35], 90.6% in dermatology [37], 91.3% in psoriatic arthritis [17], 94.6% in hematology [38], 96.3% in otolaryngology [19], and 100% in hepatology [39] and esophageal cancer [40]. Additionally, the mean annual payment of $29,000 was higher than those to CPG authors in most previously reported specialties [15,31,35-37,40]. It was also previously found that the mean annual personal payments to general rheumatologists were $4,882 to $5,673 [23]. These findings support the author’s hypothesis that pharmaceutical companies concentrate their payments significantly on rheumatology CPG authors in Japan, even compared to other specialties.

This study also evaluated the strength and quality of the CPG recommendations. A significant majority (81.8%) of the recommendations were supported by low- or very-low-quality evidence. This is particularly concerning given that the majority of strong recommendations were supported by low-quality evidence, such as case reports and opinions of experts with insufficient COI management, as seen in previous studies [3,18,32]. The incongruence between the strength of recommendations and the quality of evidence underscores the need for more rigorous evidence-based approaches in guideline development in Japan.

Nevertheless, these financial relationships between the CPG authors and the pharmaceutical companies were managed within the JCR and not publicly disclosed to the CPG readers. Although almost all CPGs published by the US and European medical societies have publicly disclosed their authors' COI information and emphasized the importance of transparency in financial relationships [2,13,14,41], non-disclosure of financial COI among CPG authors is commonly reported in Japan [18,19,35-37,40]. In some cases, significant amounts of payments and a large number of CPG authors under-declared their COI status to the public in Japan [17,31,40]. This study highlights an urgent need for transparency and reform in the development of CPGs in Japan, particularly in the field of rheumatology. The undisclosed financial COIs and the low quality of evidence supporting the recommendations have far-reaching ethical and clinical implications. They not only compromise the integrity of the guidelines but also risk skewing treatment paradigms towards less evidence-based treatments. This is particularly relevant in a field like rheumatology, where treatment decisions have long-term consequences for patients.

Limitations

This study has some limitations. First, the cross-sectional design precludes longitudinal analysis, and the focus on a single set of guidelines may not be generalizable to other fields or countries. Second, the payment data was extracted from a secondary database maintained by a non-profit research organization, which includes nearly all personal payment data collected from each pharmaceutical company. Additionally, there are no legal regulations requiring pharmaceutical companies to accurately disclose their payments to physicians in Japan. Therefore, we cannot exclude the possibility of errors in the payment data disclosed by the companies. Third, the pharmaceutical companies did not disclose their payments for purposes other than speaking, consulting, and writing by healthcare providers at the individual provider level. Furthermore, pharmaceutical companies that did not belong to the Japan Pharmaceutical Manufacturers Association did not disclose their payments to healthcare providers. Thus, there might be unmeasured financial relationships between the CPG authors and the pharmaceutical companies.

## Conclusions

All the authors of the 2020 JCR CPG for rheumatoid arthritis received personal payments from pharmaceutical companies five years before its publication. The total amount of personal payments was more than $3.6 million. The non-disclosure of financial COIs and the low quality of evidence supporting the majority of recommendations in the CPG raise significant ethical and clinical concerns. These findings call for immediate policy interventions to enhance the transparency, integrity, and reliability of CPGs in rheumatology in Japan.
